# Targeted Therapeutic Strategies for Triple-Negative Breast Cancer

**DOI:** 10.3389/fonc.2021.731535

**Published:** 2021-10-28

**Authors:** Ying Li, Zhijun Zhan, Xuemin Yin, Shujun Fu, Xiyun Deng

**Affiliations:** ^1^ Key Laboratory of Model Animals and Stem Cell Biology in Hunan Province, Department of Pathophysiology, Hunan Normal University School of Medicine, Changsha, China; ^2^ Key Laboratory of Translational Cancer Stem Cell Research, Hunan Normal University, Changsha, China

**Keywords:** targeted therapy, triple-negative breast cancer, poly (ADP-ribose) polymerase, immune checkpoint, epigenetic modification

## Abstract

Triple-negative breast cancer (TNBC) is the most aggressive subtype of breast cancer, which is characterized by the absence of estrogen receptor (ER) and progesterone receptor (PR) expression and the absence of human epidermal growth factor receptor 2 (HER2) expression/amplification. Conventional chemotherapy is the mainstay of systemic treatment for TNBC. However, lack of molecular targeted therapies and poor prognosis of TNBC patients have prompted a great effort to discover effective targets for improving the clinical outcomes. For now, poly (ADP-ribose) polymerase (PARP) inhibitors (PARPi’s) and immune checkpoint inhibitors have been approved for the treatment of TNBC. Moreover, agents that target signal transduction, angiogenesis, epigenetic modifications, and cell cycle are under active preclinical or clinical investigations. In this review, we highlight the current major developments in targeted therapies of TNBC, with some descriptions about their (dis)advantages and future perspectives.

## Introduction

Breast cancer is the type of cancer with the best-characterized molecular classification or subtyping. Clinical therapeutic efficacies vary enormously among the different subtypes, with luminal A/B subtypes and triple-negative breast cancer (TNBC) showing the best and worst outcomes, respectively (1). For TNBC, although initially responsive to chemotherapy, which is the mainstay of systemic treatment in TNBC, resistance occurs eventually in a significant portion of patients, leading to relapse of these patients. Due to the aggressive nature and lack of defined molecular targets, the poor overall survival (OS) of metastatic TNBC has remained essentially unchanged over the past two or three decades. Generally speaking, metastatic TNBC has a median OS of approximately 13 months (2), rendering improvement of the clinical outcomes an urgent task in the management of TNBC. Fortunately, we are now seeing encouraging clinical results from molecularly targeted approaches in TNBC, which include poly (ADP-ribose) polymerase inhibition and, most recently, immune checkpoint inhibition. Other potential promising targeted therapeutic strategies that are being actively investigated for TNBC include inhibition of signaling kinases (serine/threonine- or tyrosine-type), angiogenesis, epigenetic modifications, and cell cycle. The targeted therapeutic strategies of TNBC examined in clinical and preclinical studies are summarized in **Table 1**.

**Table 1:** Overview of Potential Targeted Therapeutic Strategies for TNBC.

Targets	Agents	Phase	Main Results	Advantages	Disadvantages	References
PARP	Olaparib	I/II/III	•**The OlympiA trial:** 3-year IDFS, 3-year DDFS, and OS were significantly higher in the olaparib group. •**The TBCRC 048 trial:** The ORR was 33% in germline mutations of non-BRCA1/2 HR-related genes and 31% in somatic mutations of BRCA1/2 or other HR-related genes; the median PFS for gPALB2 and sBRCA1/2 mutation carriers were 13.3 and 6.3 months; among the gPALB2 and sBRCA1/2 mutation carriers, responses occurred in 67% of TNBC patients. •**The olaparib combination with carboplatin trial:** Hematologic toxicity was the most common AE, with 36% of patients having Grade 3 and 4 neutropenia.	•Increase efficacy of BRCA-mutated breast cancer •Improve ORR, PFS and OS when combined with chemotherapy drugs	•Not suitable for patients with defective BRCA genes •High incidence of adverse events	(3–9)
	Veliparib	II	•**The I-SPY 2 trial:** Veliparib combined with carboplatin had higher rate of pCR than standard therapy alone.			
	Iniparib	II	•**The PrECOG 0105 trial:** The mean HRD-LOH scores were higher in responders compared with non-responders in iniparib clinical trails.			
Immune checkpoints	PD1: pembrolizumab	FDA-approved	**•The KEYNOTE-522 trial:** Higher percentage of patients having grade 3 or more serious AEs in the pembrolizumab plus chemotherapy group; patients in the pembrolizumab plus chemotherapy group had a higher pCR rate, which also occurred in PDL1-positive and PDL1-negative population. •**The KEYNOTE-355 trial:** Among patients with CPS of 10 or more, median PFS was significantly prolonged in the pembrolizumab plus chemotherapy group.	•Improve the OS and PFS rate of patients •Benefit from companion diagnostic	•High incidence of adverse events •Not all patients express PDL1 •No increasing pCR in combination with chemotherapy drugs	(10–15)
	PDL1: atezolizumab, durvalumab	II/III	•**The IMpassion050 trial:** Atezolizumab combination with chemotherapy didn’t increase pCR either in the intention-to-treat population or in the PDL1-positive population; in the neoadjuvant phase, patients with Grade 3/4 or more serious AEs were increased in the atezolizumab group. •**The IMpassion130 trial:** Atezolizumab plus nab-paclitaxel prolonged PFS in both intention-to-treat population and PDL1-positive population. •**The GeparNuevo study:** Increased pCR rate was observed in both durvalumab and placebo group with higher stromal TILs or positive PDL1 expression; the pCR rate of patients with high TMB and high immune GEP or TILs was notably higher compared with patients with low TMB and low immune GEP or TILs.			
Antibody-drug conjugates	Trop2: sacituzumab govitecan	FDA-approved	•**The ASCENT trial:** In the sacituzumab govitecan group, PFS and OS were significantly prolonged and pCR rate was increased. According to the therapeutic effect, sacituzumab govitecan is recently approved for metastatic TNBC patients.	•Improve the prognosis of patients •Well tolerated	•A complex engineered	(16–18)
	LIV1: ladiratuzumab vedotin	II/III	•**A phase** I**b/**II **trial:** Ladiratuzumab vedotin was well tolerated and the combination with pembrolizumab produced a synergistic effect through immunogenic cell death that might enhance anti-PD1 activity.			
Signaling pathways	EGFR: cetuximab	II	•**A phase II study:** The ORR was 20% with cisplatin plus cetuximab and 10% with cisplatin alone; patients treated with cisplatin plus cetuximab had longer PFS than those treated with cisplatin alone.	•Inhibite tumor metastasis and induce a change from mesenchymal to epithelial phenotype •Improve PFS	•Need to combine with medication for a better effect	(19–24)
	EGFR: erlotinib	Preclinical	•**Preclinical study:** Erlotinib inhibited tumor growth and metastasis and reversed a change from mesenchymal to epithelial phenotyp.			
	PI3K: BKM120	Preclinical	**•Preclinical study:** BKM120 led to significant tumor growth inhibition in PDX models (TNBC).			
	Akt: ipatasertib	II	•**A randomized, double-blind, phase II trial:** The median PFS in the ipatasertib group was 6.2 months, compared with 4.9 months in the placebo group.			
Angiogenesis	VEGF: bevacizumab	II/III	•**The RIBBON-2 trial:** Bevacizumab-containing therapy improved median PFS from 2.7 months to 6.0 months, median OS from 12.6 months to 17.9 months, and ORR from 18% to 41% and showed a 49% response rate, median TTP of 7.2 months, and median OS of 18.3 months. •**The GeparQuinto trial:** Bevacizumab to neoadjuvant anthracycline-taxane-containing chemotherapy significantly increased the pCR rate from 27.9% to 39.3% in TNBC patients. •**The BEATRICE study:** There are no differences in 3-year IDFS and OS, in which TNBC patients received chemotherapy with or without bevacizumab. •**The CALGB 40603 trail:** Patients treated with carboplatin had higher pCR breast and pCR breast/axilla rates, while patients received bevacizumab only had higher pCR breast rate.	•Show efficacy in the patient's tumor response and/or disease control	•Detrimental side-effects, along with acquired drug-resistance	(25–31)
	VEGFR: apatinib	II	•**A multicenter phase II study:** The ORR and clinical benefit rate were 10.7% and 25.0% and median PFS and OS were 3.3 months and 10.6 months in the apatinib trial.			
Epigenetic modifications	DNMT: 5-azacytidine/AZA, decitabine/DAC	Preclinical	•**Preclinical study:** PARPi’s plus AZA/DAC increased PARPi efficacy and resulted in additional tumor inhibition in TNBC cells harboring wild-type BRCA1 compared with each drug alone.	•Increase PARPi efficacy •Enhance the effect of TNBC immunotherapy.	•Lack of adequate clinical trials	(32–37)
	HDAC: suberoylanilide hydroxamic acid (SAHA),entinostat (ENT)	Preclinical	•**Preclinical study:** ENT increased the expression of ERα and aromatase and restored the sensitization of breast cancer cells to the aromatase inhibitor letrozole. SAHA could enhance the anti-tumor effects of the PARPi olaparib in TNBC cells by regulating the expression of homologous recombination repair (HRR)-related genes and hampering DNA repair.			
Cell cycle	CDK4/6: palbociclib	I/II	•**Phase I/II clinical trials** of the safety and efficacy of CDK4/6 inhibition with or without other agents in TNBC are ongoing. •**Preclinical study:** Dual blockade of PI3K and CDK4/6 had synergistic effect and could generate immunogenic cell death in TNBC cells; palbociclib could improve the sensitivity of Rb-positive TNBC cells to paclitaxel.	•Be benefited from high expression levels of mitotic checkpoint molecules in TNBC •Sufficient evidence from preclinical trials	•Not significant for patients lacking functional Rb protein •Lack of adequate clinical trials	(38–48)
	TTK: BOS172722	Preclinical	•**Preclinical study:** TTK inhibitors have anti-proliferative effects; combination of BOS172722 and paclitaxel results in significant tumor regression compared with either drug alone.			
	PLK4: CFI-400945	Preclinical	**•Preclinical study:** CFI-400945, in combination with radiation, exhibited a synergistic anti-cancer effect in TNBC cell lines and patient-derived organoids and led to a significant increase in survival to tumor endpoint in xenograft models.			
	ATR: VX-970	Preclinical	**•Preclinical study:** ATR or CHK1 inhibitor could delay the radiation-induced DNA repair and inhibit cell survival in TNBC cells.			
	CHK1: MK-8776	Preclinical				
	WEE1: MK-1775	Preclinical	**•Preclinical study:** WEE1 inhibition could overcome cisplatin resistance in TNBC cells.			
	TRAIL receptor agonist: drozitumab	Preclinical	**•Preclinical study:** TRAIL receptor agonist could induce apoptosis in TNBC cells that expressed vimentin and Axl.			

## Inhibition of Poly (ADP-ribose) Polymerases in TNBC

Poly (ADP-ribose) polymerases (PARPs) are a family of proteins involved in DNA damage repair and multiple other cellular processes. So far, 17 PARP members have been identified in human (49). Among them, PARP1 is the best-characterized family member and is responsible for 85-90% of the total PARP activity. It is activated by single-strand breaks (SSBs), thus catalyzing the synthesis of poly (ADP-ribose) chains that serve as a signal and platform to recruit other DNA repair proteins. Failure to repair SSBs because of the PARP deficiency or inhibition leads to the formation of double-strand breaks (DSBs).

**Figure 1:**
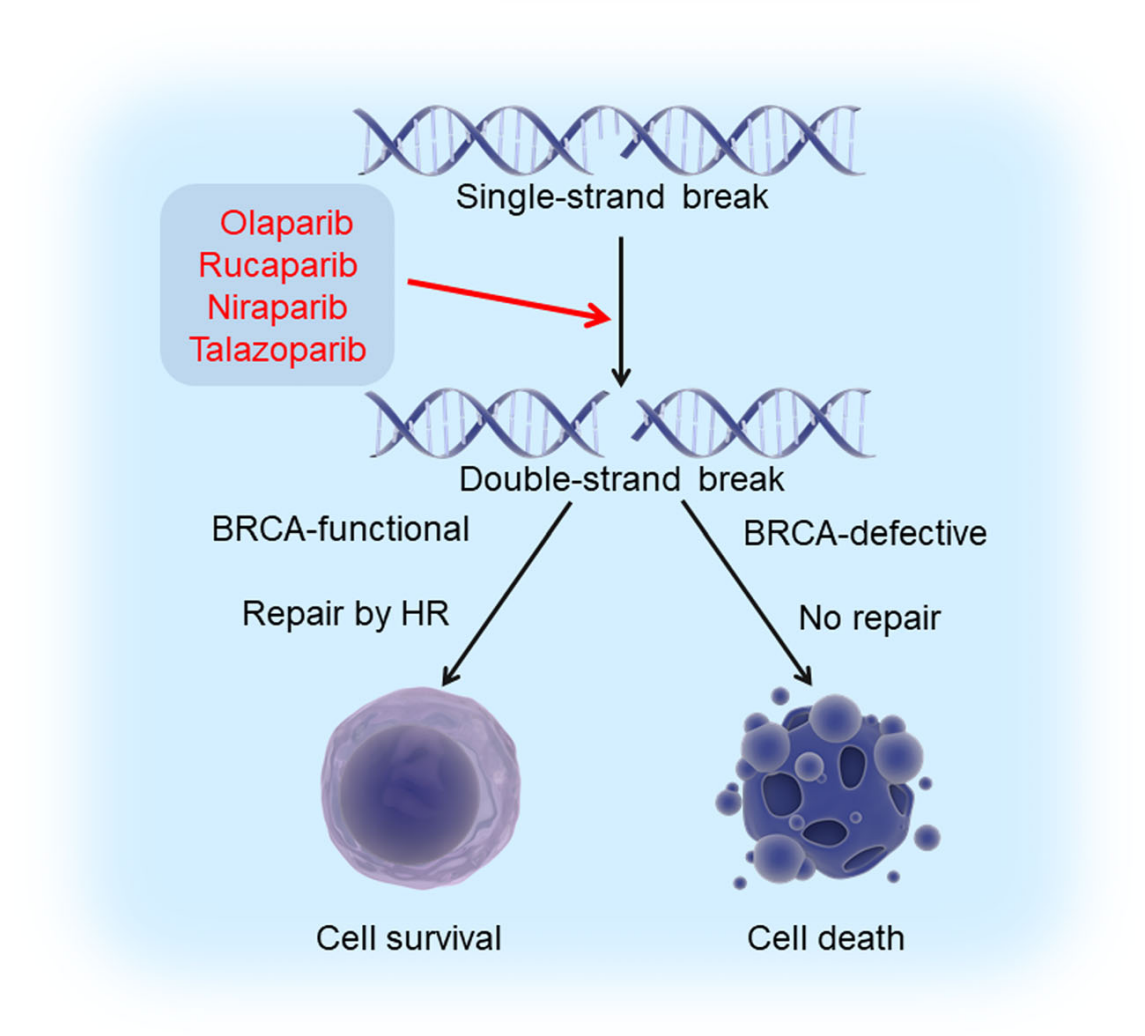
Synthetic lethality induced by PARPi’s and BRCA deficiency. Exposure of cells to PARPi’s (olaparib, rucaparib, niraparib, and talazoparib) leads to the formation of double strand breaks (DSBs) from single strand breaks (SSBs). Cells with intact BRCA function could survive since these breaks can be repaired by homologous recombination, while those with defective BRCA die because DSBs cannot be repaired. This phenomenon is known as “synthetic lethality”.

In the cells that are functional for breast cancer susceptibility gene products (BRCA1 and BRCA2), DSBs can be repaired by a process called homologous recombination (HR). Therefore, BRCA-mutated tumors are more sensitive to inhibition of PARPs due to combined loss of PARP and HR repair, an effect called “synthetic lethality” (50, 51). In the presence of PARP inhibitors (PARPi’s), the cells with BRCA defects cannot repair the DNA damage and die, whereas the cells with functional BRCAs could perform effective DNA damage repair and survive (**Figure 1**). Up to 80% of ER/PR-negative breast cancers have reduced or undetectable BRCA1 expression (52). Although germline mutations in BRCA1/2 are generally low, these mutations can confer a lifetime risk of up to 85% of developing breast cancer, with the majority (around 90%) of these tumors being triple-negative (53). Therefore, TNBC can theoretically be treated by a strategy of synthetic lethality that is based on PARP inhibition in BRCA-mutated tumors.

### PARP Inhibitors and Clinical Trials in TNBC

Various PARPi’s, which hamper DNA repair by blocking PARP-mediated PARylation, have been developed to induce synthetic lethality. Up to now, four PARPi’s, i.e., olaparib, rucaparib, niraparib, and talazoparib, have been approved by the US Food and Drug Administration (FDA) for cancer treatment. Two of them, olaparib and talazoparib, have been approved for BRCA-mutated metastatic breast cancer (54). While PARPi’s have been evaluated in clinical trials for TNBC as monotherapies, combination of PARPi’s with DNA-damaging chemotherapy appears to be a more promising approach due to increased efficacy of PARPi’s in BRCA-mutated breast cancer patients.

Olaparib is effective for patients with germline/somatic BRCA mutation or other HR-related gene mutations. The phase III OlympiA trial (NCT02032823) accessed olaparib treatment in HER2-negative breast cancer patients with germline BRCA1/2 mutations who had received neoadjuvant or adjuvant chemotherapy. The 3-year invasive disease-free survival (IDFS), 3-year distant disease-free survival (DDFS), and OS were significantly higher in the olaparib group than in the placebo group (3).

The phase II TBCRC 048 trial (NCT03344965) assessed olaparib response in metastatic breast cancer patients with germline mutations of non-BRCA1/2 HR-related genes (cohort 1) and somatic mutations of BRCA1/2 or other HR-related genes (cohort 2). The objective response rate was 33% in cohort 1 and 31% in cohort 2. Confirmed responses were only seen in patients with gPALB2 or sBRCA1/2 mutations. The median progression-free survival (PFS) for gPALB2 and sBRCA1/2 mutation carriers were 13.3 and 6.3 months, respectively. Among the gPALB2 and sBRCA1/2 mutation carriers, responses occurred in 67% of TNBC patients. No responses were observed with ATM or CHK2 mutations alone (4). This study revealed that patients with mutations of other HR-related genes might benefit from PARP inhibition.

With no BRCA mutation, patients would benefit more from combination treatment with chemotherapy and olaparib. A phase I study (NCT01445418) investigated olaparib combined with carboplatin in metastatic or recurrent TNBC patients with no germline BRCA mutation or with BRCAPro scores < 10% and negative family history. The objective response rate was 22%, with 1 patient having complete response. Hematologic toxicity was the most common adverse event (AE), with 36% of patients having Grade 3 and 4 neutropenia (5).

Veliparib, a novel PARPi that has favorable toxicity profile but is not FDA-approved yet, has been extensively studied in combination with various chemotherapeutic drugs. In a phase I clinical trial, the combination of veliparib with cisplatin and vinorelbine (a microtubule-destabilizing agent) gave rise to an overall response rate (ORR) of 73% in TNBC patients with mutated BRCA1/2 (55). In a phase III trial (NCT02032277), veliparib has been combined with paclitaxel plus carboplatin for the treatment of TNBC in standard neoadjuvant chemotherapy (56).

### Homologous Recombination Deficiency (HRD) as the Predictive Biomarker for PARP Inhibitors

BRCA1/2 and other HR-related gene mutations could cause a defect in DSB repair called homologous recombination deficiency (HRD), leading to genomic instability and thus enhanced sensitivity to PARPi’s. Therefore, HRD status (including but not limited to BRCA1/2 mutations) could be evaluated to predict the response of PARPi’s (5, 57, 58).

I-SPY 2 trial (NCT01042379) showed that the PARPi veliparib combined with carboplatin had higher rate of pathological complete response (pCR) than standard therapy alone, specifically in TNBC (6). Further study revealed that BRCA1/2 mutation carriers were more likely to achieve a pCR compared to wild-type patients in the veliparib/carboplatin arm (7). In the PrECOG 0105 (NCT00813956) trial, patients with TNBC were treated with iniparib and chemotherapy, and the mean homologous recombination deficiency loss of heterozygosity (HRD-LOH) scores were higher in responders compared with non-responders (8). Jiang et al. reported that TNBC patients with higher HRD scores might have better prognosis and benefit from DNA repair inhibitors (9).

The above studies suggest that PARPi’s have shown great promise in TNBC patients and may be used as an effective therapeutic strategy for the treatment of BRCA-mutated or even BRCA-intact TNBC. Further more excited clinical findings are expected with the optimization of the therapeutic regimen.

## Inhibition of Immune Checkpoints in TNBC

Recently, there is enormous interest in cancer immunotherapy, particularly immune checkpoint-based immunotherapy. This is demonstrated by the awarding of the Nobel Prize in Physiology or Medicine in 2018 to James P. Allison at the University of Texas MD Anderson Cancer Center and Tasuku Honjo at Kyoto University, for their seminal work in identification of immune checkpoint molecules, i.e., programmed cell death-1 (PD1), programmed death-ligand 1 (PDL1), and cytotoxic T lymphocyte-associated protein 4 (CTLA4).

### Immune Checkpoint Inhibitors and Clinical Trials in TNBC

**Figure 2:**
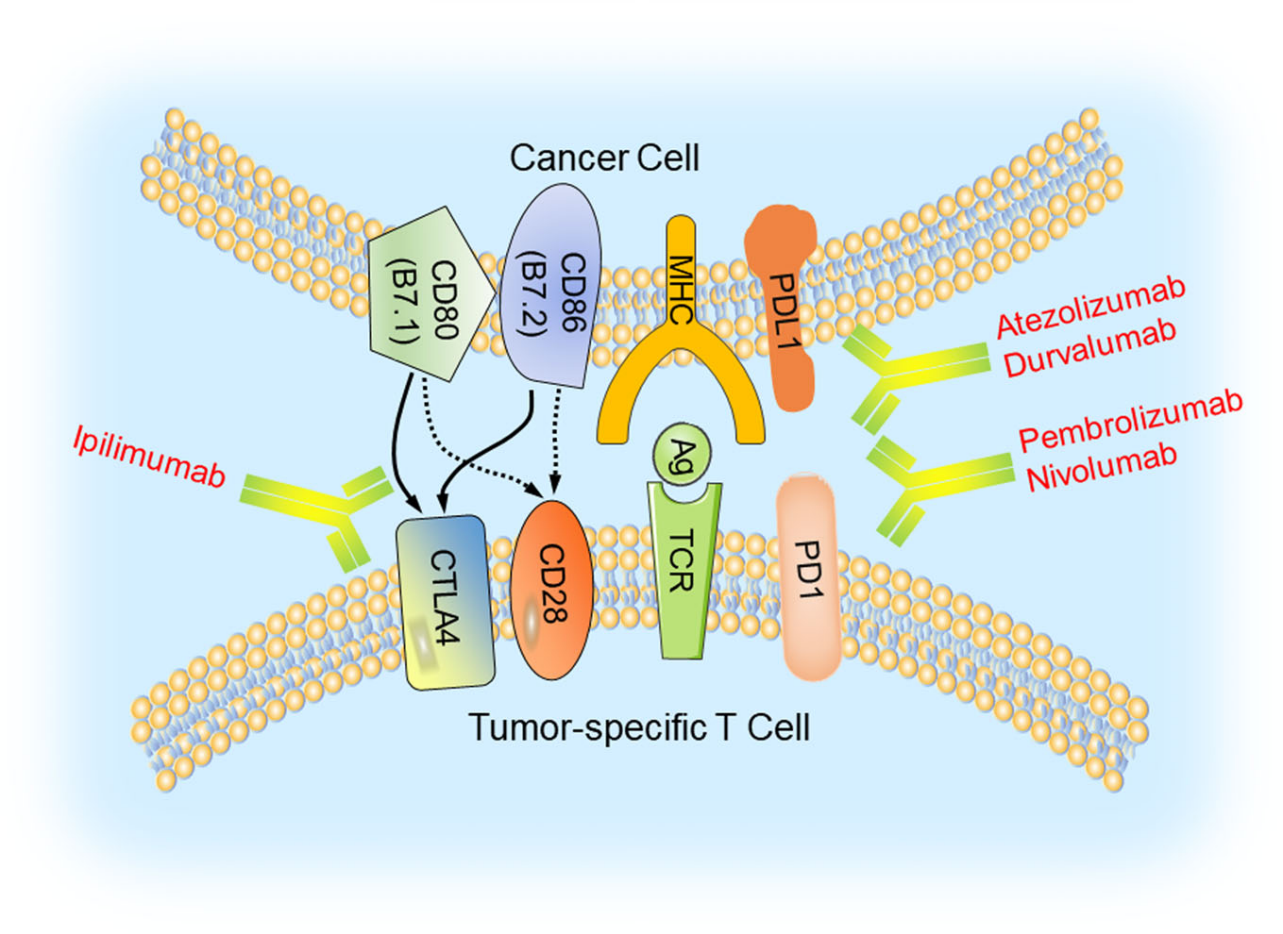
Immune checkpoint blockade in TNBC. Major histocompatibility complexes (MHCs) are antigens on the surface of the cancer cell for recognition by the cytotoxic T lymphocyte (CTL) *via* the TCR. The binding of PD1 on the surface of the CTL with its ligand PDL1 functions to suppress the activation of the CTL, leading to its cell death. CTLA4 is another inhibitory immune checkpoint molecule expressed on CTL. Antibodies (anti-CTLA4/ipilimumab, anti-PD1/pembrolizumab and nivolumab, anti-PDL1/atezolizumab and durvalumab) inhibit these immune checkpoint proteins to restore the activity of CTLs and kill cancer cells.

The discovery of these molecules led to the development of several FDA-approved humanized antibodies, so called immune checkpoint inhibitors, such as nivolumab, atezolizumab, and ipilimumab. These antibodies have demonstrated very well documented benefit for a variety of cancers (59) (**Figure 2**). Breast cancer, in general, is not an immunologically highly active cancer. However, the TNBC subtype shows higher presence of tumor-infiltrating lymphocytes (TILs) and is likely to respond to immunotherapy (60).

KEYNOTE-522 trial (NCT03036488) evaluated the safety and efficacy of pembrolizumab plus chemotherapy as neoadjuvant therapy, followed by definitive surgery and pembrolizumab as adjuvant therapy in patients who had early TNBC. Most treatment-related AEs occurred during the neoadjuvant phase, with higher percentage of patients having grade 3 or more serious AEs in the pembrolizumab plus chemotherapy group than in the placebo plus chemotherapy group. Consistent results were observed in the adjuvant phase. At the first and second interim analysis, patients in the pembrolizumab plus chemotherapy group had a higher pCR rate, which also occurred in PDL1-positive and PDL1-negative population, indicating that PDL1 expression was not a suitable predictor of response in early TNBC (10).

IMpassion050 trial (NCT03726879) evaluated the efficacy and safety of atezolizumab compared with placebo when it was combined with chemotherapy in high risk, HER2-positive early breast cancer. Results showed that this combination didn’t increase pCR either in the intention-to-treat population or in the PDL1-positive population. In the neoadjuvant phase, patients with Grade 3/4 or more serious AEs were increased in the atezolizumab group. There were 4 patients with Grade 5 AEs, including alveolitis, septic shock, sepsis, and COVID-19, in the neoadjuvant phase and 1 patient in the adjuvant phase (11).

### PDL1 Expression, Tumor Mutation Burden (TMB), and Immune Infiltration as Predictive Biomarkers of Immune Checkpoint Inhibitors

Clinical trials have shown a correlation between high expression of PDL1 and efficacy of immune checkpoint inhibitors in metastatic TNBC. Thus, PDL1 could be a potential predictive biomarker of response to immunotherapy. Two antibody-based companion diagnostics for PDL1 expression are available. The PDL1 IHC 22C3 pharmDx (Agilent Technologies) is approved for selecting patients for treatment with pembrolizumab, using a cutoff of combined positive score (CPS) of 10. The Ventana PDL1 (SP142) assay (Roche Diagnostics) is approved for treatment with atezolizumab in metastatic TNBC, using a cutoff of immune cell (IC) score of 1% (61, 62).

In the phase III KEYNOTE-355 trial (NCT02819518), metastatic TNBC patients were randomly assigned 2:1 to receive pembrolizumab plus chemotherapy or placebo plus chemotherapy. PDL1 expression of formalin-fixed tumor samples was assessed by the PDL1 IHC 22C3 pharmDx assay and characterised by CPS. Among patients with CPS of 10 or more, median PFS was significantly prolonged in the pembrolizumab plus chemotherapy group (12).

In the IMpassion130 trial (NCT02425891), patients with untreated metastatic TNBC were randomly assigned in a 1:1 ratio to receive PDL1 antibody atezolizumab plus nab-paclitaxel or placebo plus nab-paclitaxel. The PDL1 expression on tumor-infiltrating immune cells was evaluated by PDL1 (SP142) immunohistochemical assay (IC score ≥ 1%, PDL1-positive). Kaplan-Meier analysis showed that atezolizumab plus nab-paclitaxel prolonged PFS in both intention-to-treat population and PDL1-positive population (13).

Besides PDL1 expression, tumor mutation burden (TMB) and immune infiltration could also be predictors for immune checkpoint inhibitor response. In the phase II GeparNuevo study (NCT02685059), patients with early TNBC were randomly assigned to receive durvalumab or placebo in addition to chemotherapy. Increased pCR rate was observed in both durvalumab and placebo group with higher stromal TILs or positive PDL1 expression (14). Whole exome sequencing and RNA sequencing of these samples showed that median TMB was significantly higher in patients with a pCR. The pCR rate of patients with high TMB and high immune gene expression profile (GEP) or TILs was notably higher compared with patients with low TMB and low immune GEP or TILs, which indicated both TMB and immune GEP or TILs were pCR predictors (15).

These findings are expected to lead to new effective treatment options for patients with TNBC. The immune checkpoint-based strategy for the therapy of TNBC is the topic of our recently published review. For sake of saving time and space, immune checkpoint inhibition in TNBC will not be described redundantly here. Interested readers please refer to our review (63) and another review published last year by Keenan et al. (64).

It should be noted that the benefit of immune checkpoint inhibition in TNBC is dependent on the protein level of the immune checkpoint molecules. For example, patients with PDL1-positive immune cells had prolonged PFS treated with atezolizumab (13). Furthermore, the status of post-translational modifications such as glycosylation of the PDL1 protein also significantly impacts the therapeutic efficacy of immune checkpoint inhibition in TNBC (65). For patients with unresectable locally advanced or metastatic TNBC whose tumors have PDL1 expression ≥ 1%, atezolizumab plus nab-paclitaxel is an effective therapeutic option (66). Therefore, it will be pivotal to screen predictors of response to immune checkpoint inhibitors for better option. In addition, combination with chemotherapy would benefit more than immune checkpoint inhibition alone.

## Application of Antibody-Drug Conjugates in TNBC

Antibody-drug conjugates (ADCs) are complex engineered therapeutics composed of monoclonal antibodies that specifically recognize tumor-associated antigens and cytotoxic agents that bind to the antibody *via* a linker. ADCs could precisely target the cells and are internalized through endocytosis. Then they are decomposed to release cytotoxic agents, which induce cell death eventually. This targeted therapeutic delivery approach could reduce off-target toxicity by limiting normal tissues exposed to the cytotoxic agents (67).

Sacituzumab govitecan comprises an antibody targeting trophoblast cell-surface antigen 2 (Trop2), which couples to SN-38, a topoisomerase I inhibitor, through cleavable CL2A linker. A phase III ASCENT trial (NCT02574455) evaluated the efficacy of sacituzumab govitecan comparing with single-agent chemotherapy in patients with relapsed or refractory metastatic TNBC. In the sacituzumab govitecan group, PFS and OS were significantly prolonged and pCR rate was increased (16). According to the therapeutic effect, sacituzumab govitecan is recently approved for metastatic TNBC patients who have received two prior lines of therapy.

Ladiratuzumab vedotin (or SGN-LIV1A) is an investigational anti-LIV1 antibody-drug conjugate. The antibody binds to monomethyl auristatin E *via* a protease-cleavable linker. A phase Ib/II trial (NCT03310957) studied the combination of ladiratuzumab vedotin with pembrolizumab in patients with metastatic TNBC. Preliminary results showed ladiratuzumab vedotin was well tolerated and the combination with pembrolizumab produced a synergistic effect through immunogenic cell death that might enhance anti-PD1 activity (17, 18).

## Inhibition of Signaling Pathways in TNBC

**Figure 3:**
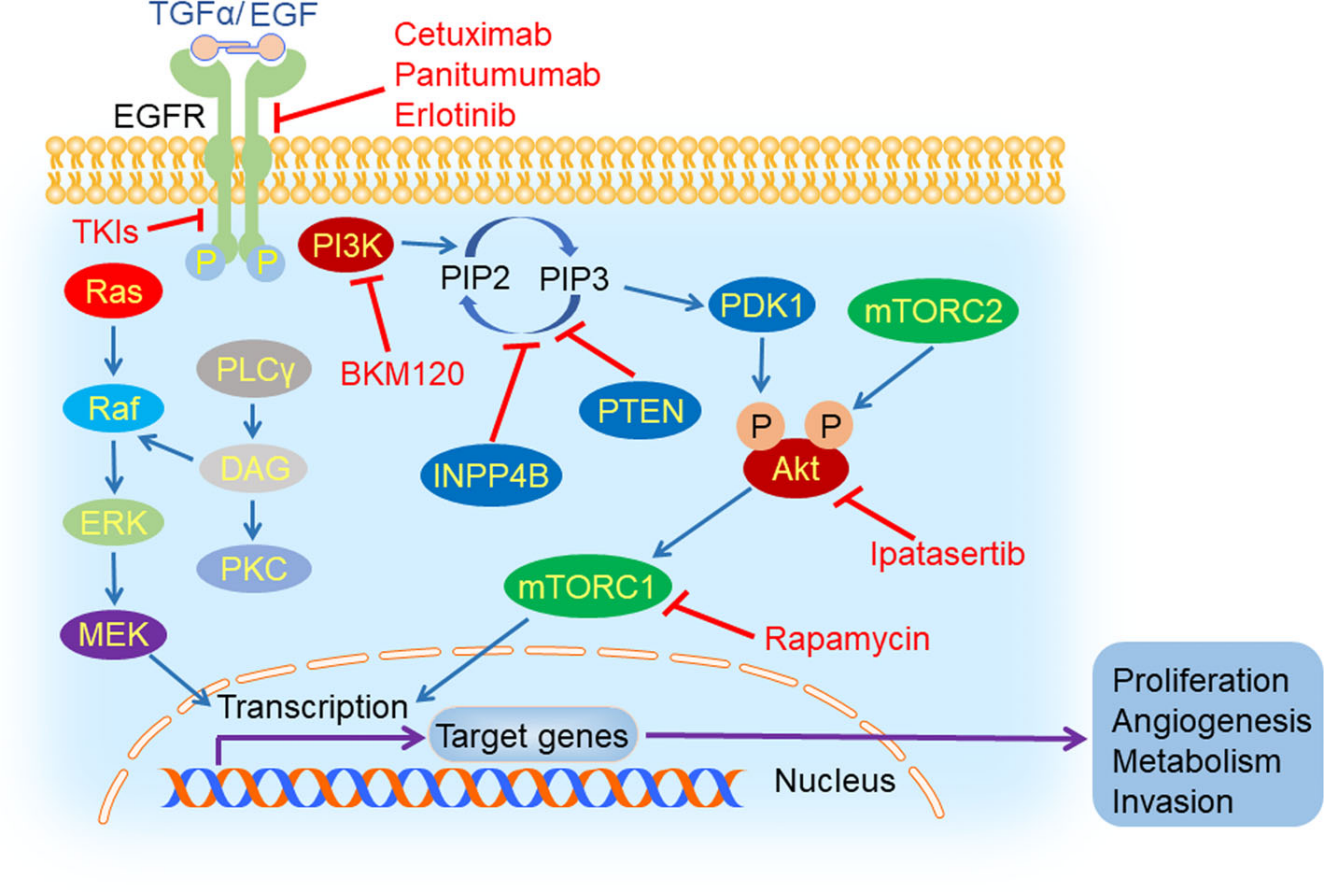
EGFR and its downstream signaling pathways inhibition in TNBC. Epidermal growth factor receptor (EGFR) could be activated by its ligand EGF or transforming growth factor α (TGFα). After its activation, it can dimerize with all members of HER family and create homo- or hetero-dimers, triggering a myriad of downstream signaling pathways, such as PI3K/Akt/mTOR, Ras/Raf/MEK/ERK and PLCγ/PKC. EGFR inhibitors (cetuximab, TKIs, panitumumab, and erlotinib), PI3K inhibitor (BKM120), mTORC1 inhibitor (rapamycin) and Akt inhibitor (ipatasertib) could hamper tumorigenesis and tumor progression by suppressing the process of signal transduction.

In cancer cells, some signaling pathways are highly activated, such as EGFR and its downstream PI3K/Akt/mTOR pathway (**Figure 3**), which could accelerate tumor initiation and progression. Thus, inhibiting these signaling pathways might be a potential therapeutic strategy for TNBC patients.

### EGFR Inhibition

Epidermal growth factor receptor (EGFR) is a glycoprotein located on the surface of the cell membrane, which belongs to the HER family of transmembrane receptors. EGFR is activated by binding to its ligand including epidermal growth factor (EGF) and transforming growth factor α (TGFα). Following ligand binding, it can dimerize with all members of the HER family and generate homo- or hetero-dimers which could be autophosphorylated (68). The autophosphorylation triggers a myriad of downstream signaling pathways, such as PI3K/Akt, Ras/Raf/MEK/ERK and PLCγ/PKC, that play an important role in cell survival, proliferation, differentiation, motility, apoptosis, migration, adhesion, and angiogenesis (69). In TNBC, EGFR was overexpressed and was closely related with carcinogenesis and tumor progression (70). The expression of EGFR was negatively correlated with prognosis of TNBC patients (71).

EGFR could be targeted by monoclonal antibodies (cetuximab, panitumumab) and tyrosine kinase inhibitors (TKIs). Monoclonal antibodies and TKIs are approved for the treatment of advanced cancers, such as colorectal cancers and non-small cell lung cancers (72). However, two randomized phase II trials targeting EGFR in TNBC have not demonstrated significant beneficial effects. In the TBCRC 001 study, metastatic TNBC patients were treated with cetuximab alone and then plus carboplatin in progression compared to the combination therapy from the beginning. In another phase II study (NCT00463788), patients with metastatic TNBC received cisplatin plus cetuximab or cisplatin alone. The ORR was 20% with cisplatin plus cetuximab and 10% with cisplatin alone. Patients treated with cisplatin plus cetuximab had longer PFS than those treated with cisplatin alone (19).

Despite the unsatisfactory clinical data, the results should not be ignored when considering the potential of anti-EGFR agents in TNBC. A preclinical study from MD Anderson Cancer Center demonstrated that the EGFR tyrosine kinase inhibitor erlotinib inhibited tumor growth and metastasis and reversed a change from mesenchymal to epithelial phenotype by increasing the expression of E-cadherin and decreasing the expression of vimentin in TNBC cells (20). Another preclinical research showed that erlotinib inhibited tumor growth and metastasis in a SUM149 xenograft mouse model, which might be non-specific effect of EGFR inhibition since erlotinib could inhibit other kinases (21). The above results suggest that EMT modulation by targeting EGFR may reduce metastasis of TNBC, and inhibiting EGFR may be a potential therapeutic approach to patients with TNBC.

### PI3K/Akt/mTOR Inhibition

Phosphoinositide 3-kinase (PI3K) is a lipid kinase which is activated by receptor tyrosine kinases (RTKs) and catalyzes phosphatidylinositol 4,5-bisphosphate (PIP_2_) to inositol 1,4,5-trisphosphate (IP_3_) subsequently. Phosphoinositide-dependent kinase 1 (PDK1) and Akt are both recruited by IP_3_ and located near the plasma membrane. Then, Akt is phosphorylated at Thr308 by PDK1, leading to its partial activation. Full activation of Akt occurs upon the phosphorylation at Ser473 by mTORC2 (73). The PI3K/Akt/mTOR signaling pathway plays a vital role in cell growth, proliferation, angiogenesis, and metabolism (74), which is negatively regulated by PTEN and INPP4B (75).

The PI3K/Akt/mTOR pathway is an important oncogenic driver in TNBC. The activation mutations of PIK3CA, the gene encoding the catalytic subunit of PI3K (76), are 23.7% in TNBC. The inhibition of the PI3K/Akt/mTOR signaling pathway has exhibited a promising prospect in treating TNBC. In patient-derived xenograft (PDX) models originating from TNBC, the PI3K inhibitor BKM120 was used to evaluate their response by measuring tumor growth. It has been shown that BKM120 therapy led to significant tumor growth inhibition in all models, with the percentage of tumor growth inhibition (%TGI) ranging from 35% in the least sensitive model WHIM12 (PTEN-deficient) and 84% in the most sensitive model WHIM4 (PTEN-normal) (22). Lin et al. proposed another strategy for using an mTORC1 inhibitor, rapamycin, to combat metastatic TNBC with upregulated Gαh, also known as tissue transglutaminase (tTG) or transglutaminase 2 (TG2) (23). Patients from a randomized, double-blind, phase II trial (NCT02162719) received intravenous paclitaxel with or without Akt inhibitor ipatasertib until disease progression or unacceptable toxicity. Results showed that median PFS in the ipatasertib group was 6.2 months, compared with 4.9 months in the placebo group. These are the first results supporting Akt-targeted therapy for TNBC (24). The development of drugs targeting the PI3K/Akt/mTOR pathway for the treatment of TNBC is an emerging field, and we look forward to more promising clinical trials.

## Inhibition of Angiogenesis in TNBC

**Figure 4:**
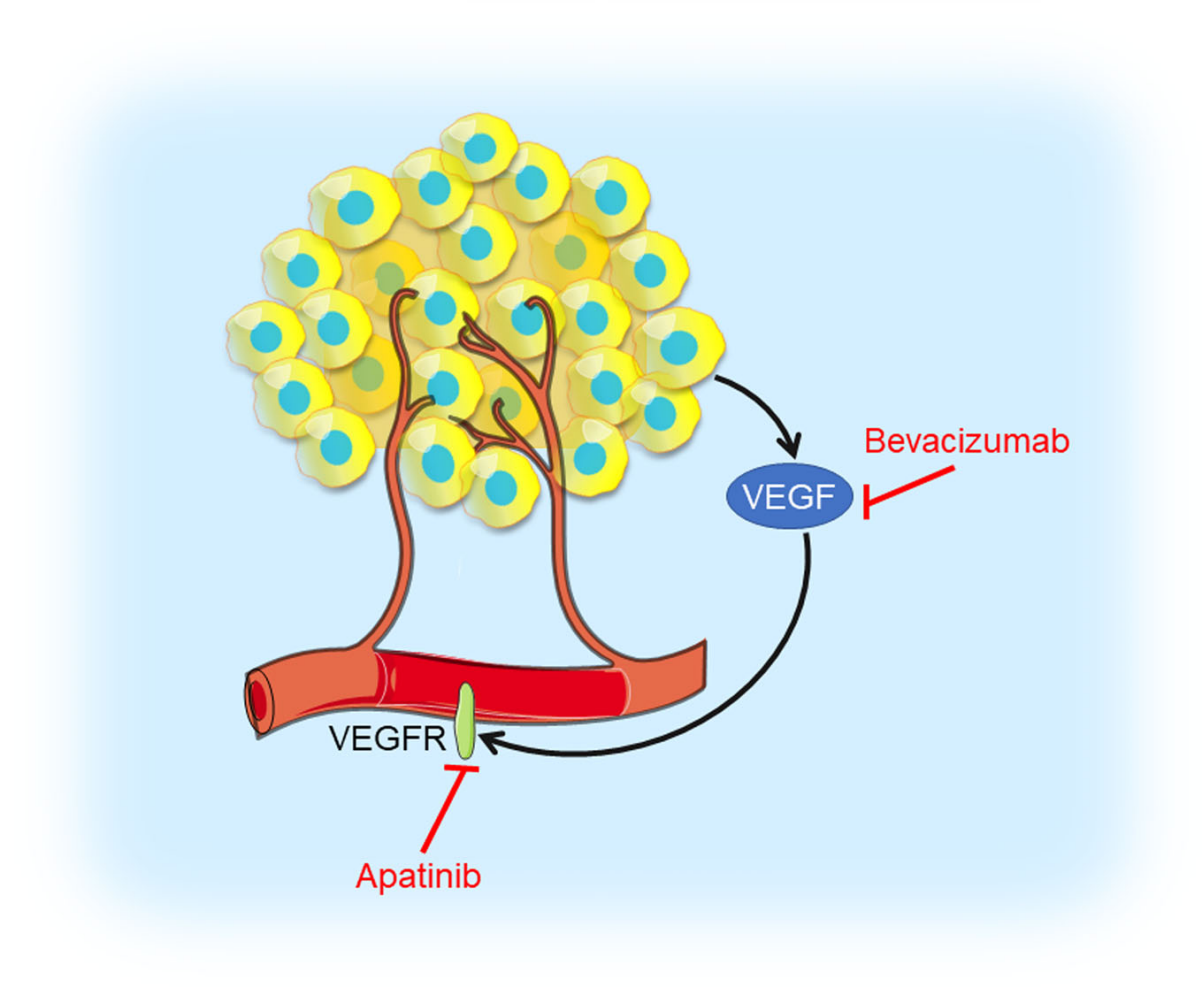
Angiogenesis inhibition in TNBC. Tumor cells produce VEGF which interacts with VEGFR contributing to angiogenesis. VEGF inhibitor bevacizumab and VEGFR inhibitor apatinib could prevent VEGF interacting with VEGFR, thus blocking tumor angiogenesis.

Solid tumors couldn’t grow beyond a certain size or metastasize to another organ without blood vessels (77). Thus, blocking tumor angiogenesis could cut off intertumoral oxygen and nutritional supply and arrest tumor growth (**Figure 4**). Vascular endothelial growth factor (VEGF) and its receptor VEGFR have been demonstrated to be major contributors to angiogenesis (78). The VEGF signaling stimulates cellular pathways that promote the formation of intertumoral blood vessels, leading to rapid tumor growth and metastatic potential (79).

VEGF is highly expressed in TNBC and a higher VEGF content is significantly correlated with shorter relapse-free survival (RFS) as well as OS (80). Bevacizumab is a humanized antibody binding to VEGF-A, the prototype VEGF family member, which prevents VEGF from interacting with its receptor, VEGFR. A randomized phase III RIBBON-2 trial revealed that second-line bevacizumab-containing therapy for TNBC patients improved median PFS from 2.7 months to 6.0 months, median OS from 12.6 months to 17.9 months, and ORR from 18% to 41%, respectively (25). A first-line bevacizumab-containing therapy showed a 49% response rate, median time to progression (TTP) of 7.2 months, and median OS of 18.3 months, respectively, for metastatic TNBC (26). In the GeparQuinto trial indicated the addition of bevacizumab to neoadjuvant anthracycline-taxane-containing chemotherapy significantly increased the pCR rate from 27.9% to 39.3% in TNBC patients (27). Results from a phase II neoadjuvant trial showed bevacizumab combined with docetaxel and carboplatin as neoadjuvant chemotherapy resulted in an encouraging pCR rate (42%) in TNBC (28). However, no differences in 3-year invasive disease-free survival (IDFS) and OS were noted in a phase III BEATRICE study (NCT00528567), in which TNBC patients received chemotherapy with or without bevacizumab (29). Moreover, in CALGB 40603 trail (NCT00861705), the efficacy of carboplatin or bevacizumab combined neoadjuvant chemotherapy were evaluated in stage II to III TNBC. Patients treated with carboplatin had higher pCR breast and pCR breast/axilla rates, while patients received bevacizumab only had higher pCR breast rate. Those received both agents had the highest pCR rate, with no significant interaction between their effects (30). A multicenter phase II study (NCT01176669) of VEGFR inhibitor apatinib treating metastatic TNBC patients revealed that the ORR and clinical benefit rate were 10.7% and 25.0%, respectively. Median PFS and OS were 3.3 months and 10.6 months, respectively (31). These angiogenesis inhibitors have shown objective efficacy in clinical trials of TNBC and had controllable toxicity, but testing in breast cancer that is highly angiogenesis-dependent might provide more convincing evidence for novel strategy of TNBC treatments.

## Inhibition of Epigenetic Modifications in TNBC

**Figure 5:**
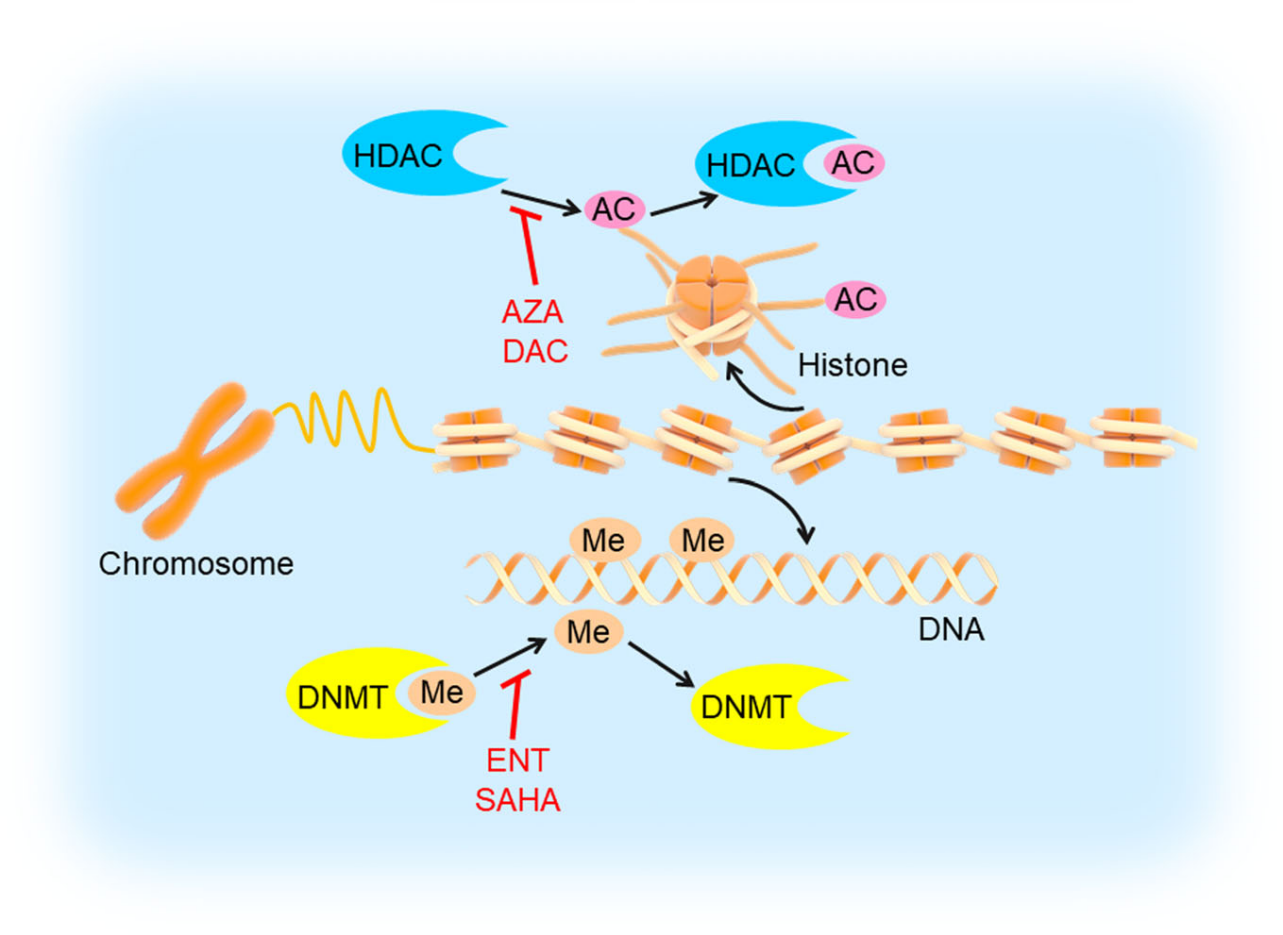
DNA methylation and histone deacetylation inhibition in TNBC. DNA is methylated by DNMT and histone is deacetylated by HDAC, which could be inhibited by DNMTi’s (entinostat/ENT, suberoylanilide hydroxamic acid/SAHA) and HDACi’s (5-azacytidine/AZA, decitabine/DAC), respectively. This would induce tumor cell apoptosis and inhibit angiogenesis, cell migration and invasion.

Epigenetic modifications often specify stably heritable changes in phenotype resulting from changes in a chromosome without alterations in the DNA sequence (81). With decades of research, epigenetic modifications have emerged as fundamental players in cancer development and progression, which mainly include DNA modifications (such as DNA methylation) and histone modifications (such as histone deacetylation) (**Figure 5**) (82). DNA methylation recruits proteins involved in gene repression or inhibits the binding of transcription factors to DNA to regulate gene expression (83). Histone modifications could influence chromatin compaction and accessibility through many ways, including acetylation, phosphorylation, ubiquitinylation, and sumoylation (84). Additionally, epigenetic modifications are being developed as clinical biomarkers for diagnostic, prognostic, and therapeutic applications in tumors (85, 86). Therefore, inhibiting DNA methylation and histone deacetylation may be a probable targeted therapeutic strategy.

### DNMT Inhibition

DNA methylation refers to the process that a methyl group is added to the 5′ position of the cytosine ring in CpG dinucleotides. Tumor suppressor genes, such as BRCA1, could be inhibited in tumors by promoter hypermethylation, which may be an important mechanism of primary breast cancer progression (87, 88). A research based on the analysis of a large number of breast cancer cases confirmed that BRCA1 is abnormally methylated in sporadic tumors and methylation of BRCA1 played a key role in breast tumorigenesis. Moreover, methylation of BRCA1 is negatively correlated with ER and PR expression (89).

DNA methylation is initiated by DNA methyltransferases (DNMTs). The DNMT family enzymes consist of DNMT1, DNMT2, DNMT3A, and DNMT3B, among which DNMT1 is the crucial maintenance methyltransferase in humans (90). DNMT1 was highly expressed in TNBC compared to other subtypes. The expression of DNMT1 was negatively associated with OS in breast cancer (91). A preclinical study showed that PARPi’s plus DNMT inhibitors (DNMTi’s, 5-azacytidine/AZA, decitabine/DAC) increased PARPi efficacy and resulted in additional tumor inhibition in TNBC cells harboring wild-type BRCA1 compared with each drug alone (32). Although it was only a preclinical study in TNBC, DNMTi’s had been approved by the US FDA for treating other cancers, such as myeloid malignancies and could be promising agents for TNBC treatment (33).

### HDAC Inhibition

Histone deacetylase (HDAC) is an enzyme that deacetylates histone proteins. The deacetylation of histones leads to chromatin condensation, which ultimately represses the transcription of gene expression. The negative regulation of tumor suppressor gene is associated with tumor cell invasion, migration, proliferation, and angiogenesis. In contrast, HDAC inhibitors (HDACi’s) could reverse the gene expression suppression through histone hyperacetylation and chromatin relaxation. More specifically, HDACi’s could induce tumor cell apoptosis and inhibit angiogenesis, cell migration, and invasion (92, 93).

In a preclinical study, researchers found the HDACi entinostat (ENT) increased the expression of estrogen receptor-α (ERα) and aromatase in breast cancer cells and restored the sensitization of breast cancer cells to the aromatase inhibitor letrozole both *in vitro* and *in vivo*. These results suggested that combination of histone deacetylase and aromatase inhibitors could be used to treat ER-negative and endocrine therapy-resistant breast cancer (34). Sulaiman et al. have revealed that the expression of mTORC1 and HDAC were higher in TNBC than in luminal breast cancer. Co-inhibition of mTORC1 and HDAC with rapamycin plus valproic acid reproducibly promoted estrogen receptor 1 (ESR1) gene expression in TNBC cells (35). HDACi’s increase PDL1 and HLA-DR expression in TNBC and reduce the proportion of CD4Foxp3^+^ T cells. PD1 and CTLA4 blockade promoted TIL infiltration, cell apoptosis, and tumor regression. Thus, HDAC inhibition by HDACi’s could potentiate the tumor-suppressive effects of immunotherapy in TNBC (36). Another study has demonstrated that the HDACi suberoylanilide hydroxamic acid (SAHA) could enhance the anti-tumor effects of the PARPi olaparib in TNBC cells by regulating the expression of homologous recombination repair (HRR)-related genes and hampering DNA repair (37).

## Inhibition of Cell Cycle in TNBC

**Figure 6:**
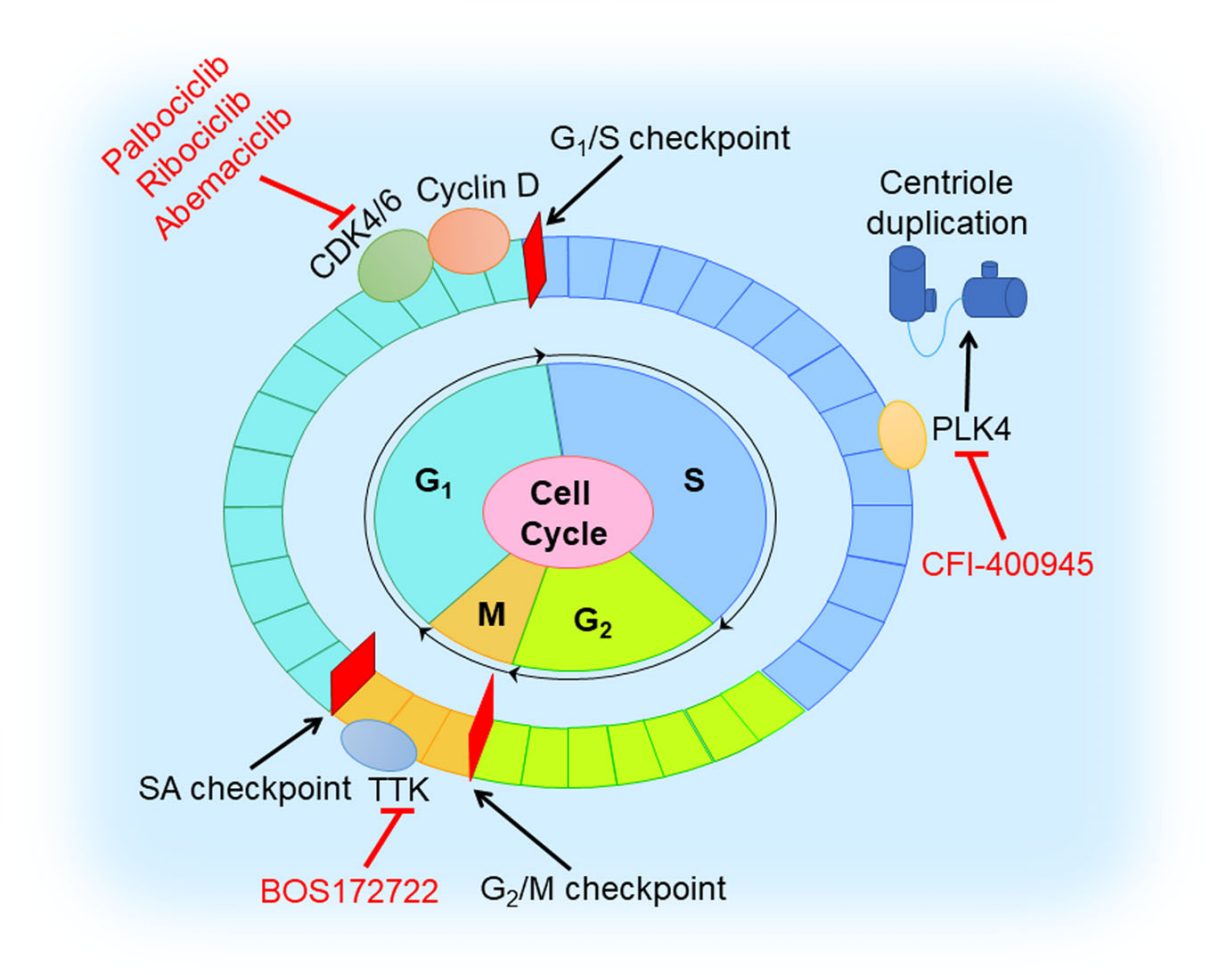
Cell cycle inhibition in TNBC. Cell cycle involves G_1_, S, G_2_, and M phases. CDK4/6 inhibitor (palbociclib, ribociclib, and abemaciclib) blocks the cell cycle at G_1_ to S transition by triggering dephosphorylation of retinoblastoma tumor suppressor protein (Rb). TTK inhibitor (BOS172722) binds to TTK that controls the spindle assembly checkpoint. PLK4 inhibitor (CFI-400945) reduces centriole duplication to prevent tumor growth.

The cell cycle involves four ordered phases denoted G_1_ (resting stage), S (DNA synthesis), G_2_ (protein synthesis), and M (mitosis) (**Figure 6**). To ensure the fidelity of the cell cycle, several checkpoints arrest cell cycle to allow cells to properly repair defects during DNA synthesis and chromosome segregation (94). Cyclin-dependent kinases (CDKs) are activated and promote cell cycle progression with binding to cyclins that are synthesized and cleared during the cell cycle (95). Tumors with dysregulated CDKs often induce unscheduled proliferation (94).

It is well-known that the CDK4/6 inhibitors, blocking the cell cycle at the G_1_ to S transition by triggering the dephosphorylation of retinoblastoma tumor suppressor protein (Rb) (96), play a vital role in preventing the proliferation of cancer cells. For now, three CDK4/6 inhibitors (palbociclib, ribociclib, and abemaciclib) received FDA approval for the treatment of HR-positive or HER2-negative breast cancer (97–100). However, the therapeutic effect of CDK4/6 inhibitors in TNBC is poor since loss of Rb often occurs. Extensive studies have revealed that combination with other molecules inhibition or therapy, such as PI3K inhibition, AR inhibition, immune checkpoint blockage, and chemotherapy, might help to overcome drug resistance in TNBC (38). In a preclinical study, dual blockade of PI3K and CDK4/6 had synergistic effect and could generate immunogenic cell death in TNBC cells (39). Pretreatment with palbociclib could improve the sensitivity of Rb-positive TNBC cells to paclitaxel (40). Phase I/II clinical trials of the safety and efficacy of CDK4/6 inhibition with or without other agents (anti-androgen medication, anti-PDL1 antibody, and chemotherapeutic drugs) in TNBC are ongoing (38).

Another class of agents targeting the cell cycle is TTK protein kinase inhibitors. TTK, namely monopolar spindle 1 (MPS1), controls the spindle assembly checkpoint (SAC) that ensures the integrity and stability of the genome in mitosis (101). TNBC has high expression levels of mitotic checkpoint molecules, and consequently, TTK inhibitors might prevent TNBC growth and proliferation (41). A preclinical trial demonstrated MPS1/TTK inhibitors have anti-proliferative effects in basal BC cell lines, with the half-maximal inhibitory concentration (IC_50_) values ranging from 0.05 to 1.0 μM (42). Anderhub et al. showed that in multiple xenograft models of human TNBC, the combination of MPS1 inhibitor BOS172722 and paclitaxel results in significant *in vivo* efficacy, showing significant tumor regression compared with either drug alone (43).

Polo-like kinase 4 (PLK4), a regulator of the centriole duplication, is crucial to the maintenance of centriole and centrosome numerical integrity. PLK4 inhibitors would potentiate aneuploidy and genomic instability and lead to cancer cell death (102). An *in vitro* experimental study showed that a novel inhibitor of PLK4, CFI-400945, in combination with radiation, exhibited a synergistic anti-cancer effect in TNBC cell lines and patient-derived organoids and led to a significant increase in survival to tumor endpoint in xenograft models *in vivo*, compared to control or single-agent treatment (44). However, overactivation of PLK4 is always correlated with centrosome amplification (CA) promoting a high risk of breast cancer (103). Further preclinical studies are warranted to characterize molecular mechanisms of action of this combination and its potential clinical applications, and lay a theoretical foundation for PLK4 to be used as a promising target in TNBC.

Beyond this, ATR, CHK1, WEE1, and TRAIL might also be targets in TNBC. Preclinical studies showed that ATR or CHK1 inhibitor could delay the radiation-induced DNA repair and inhibit cell survival in TNBC cells (45, 46), while WEE1 inhibition could overcome cisplatin resistance in TNBC cells (47), and TRAIL receptor agonist could induce apoptosis in TNBC cells that expressed vimentin and Axl (48).

## Concluding Remarks

TNBC is a complex disease with poor prognosis and rare effective targeted therapy. It is urgent to explore novel targeted therapeutic strategies. For now, PARP inhibition has shown great promise in BRCA1/2-mutated TNBC patients. It is of great hope to combine PARPi’s with DNA-damaging chemotherapy for TNBC patients harboring wild-type BRCA1/2. Meanwhile, results of clinical and preclinical studies have revealed that immunotherapy with checkpoint blockage gives rise to a good outcome in PD1/PDL1-positive TNBC patients. Targeting VEGF/VEGFR alone provides potential efficacy by inhibiting angiogenesis. However, many patients develop drug resistance while interconnected or compensatory pathways could overcome VEGF/VEGFR-targeted inhibition (78). As the “genomic medicines”, epigenetic drugs (DNMTi’s, HDACi’s, etc) have shown great application prospects in treating TNBC patients. Targeting epigenetic modifications have exhibited great efficacy when used jointly with other therapies such as chemotherapy or immunotherapy (104). CDK4/6 is the main target of cell cycle in breast cancer. When combined with other targeted therapeutic agents, CDK4/6 inhibitors could benefit more TNBC patients.

In summary, each targeted therapy in TNBC has its advantages and disadvantages when applied alone. Thus, combination of various targeted therapies would be a better strategy to enhance the therapeutic effectiveness and benefit more TNBC patients. Additionally, it is also warranted to conduct more and in-depth studies to identify novel effective therapeutic targets in TNBC. Hopefully, TNBC patients will have more individualized treatment options and better outcomes in the near future.

## Author Contributions

YL, ZZ, and XY: Reviewing the literature and writing the original manuscript. SF: Writing, revising, and editing the manuscript. XD: Revising and reviewing the manuscript. All authors contributed to the article and approved the submitted version.

## Funding

This work was supported by National Natural Science Foundation of China (82103342), Natural Science Foundation of Hunan Province (2021JJ40366), Key Grant of Research and Development in Hunan Province (2020DK2002), Changsha Municipal Natural Science Foundation (kq2014080), National Students’ Platform for Innovation and Entrepreneurship Training Program (S202010542026), and Hunan Normal University School of Medicine Open Project Fund (KF2021019).

## Conflict of Interest

The authors declare that the research was conducted in the absence of any commercial or financial relationships that could be construed as a potential conflict of interest.

## Publisher’s Note

All claims expressed in this article are solely those of the authors and do not necessarily represent those of their affiliated organizations, or those of the publisher, the editors and the reviewers. Any product that may be evaluated in this article, or claim that may be made by its manufacturer, is not guaranteed or endorsed by the publisher.
